# A Novel ER Stress Mediator TMTC3 Promotes Squamous Cell Carcinoma Progression by Activating GRP78/PERK Signaling Pathway

**DOI:** 10.7150/ijbs.72838

**Published:** 2022-07-18

**Authors:** Hongyu Yuan, Zitong Zhao, Zichan Guo, Liying Ma, Jing Han, Yongmei Song

**Affiliations:** 1 State Key Laboratory of Molecular Oncology, National Cancer Center/National Clinical Research Center for Cancer/Cancer Hospital, Chinese Academy of Medical Sciences and Peking Union Medical College, Beijing 100021, China.; 2 Department of Medical Oncology, Hebei Medical University Fourth Affiliated Hospital and Hebei Provincial Tumor Hospital, Shijiazhuang, Hebei 050000, China.

**Keywords:** TMTC3, squamous cell carcinoma, EMT, ER stress

## Abstract

During tumor progression, tumor cells are exposed to various stress conditions, which result in endoplasmic reticulum (ER) stress and activate the unfolded protein response (UPR) to restore ER homeostasis. Accumulating evidence reported the orchestrating role of ER stress in epithelial-mesenchymal transition (EMT) progress, but the detailed mechanism was unclear. Here, we identified ectopic expression of TMTC3 in cells undergoing ER stress and verified the association with EMT markers through the cellular model of ER stress and database analysis. TMTC3 was abnormally highly expressed in squamous cell carcinomas (SCCs), and regulated by TP63, an SCCs-specific transcription factor. Biological function experiments indicated that TMTC3 promoted a malignant phenotype *in vitro*, and accelerated tumor growth and metastasis *in vivo*. RNA-seq analyses and further experiments revealed that TMTC3 promoted the expression of EMT markers via interleukin-like EMT inducer (ILEI, FAM3C). Further studies on the mechanism showed that TMTC3 disrupted the interaction between PERK and GRP78 to activate the PERK pathway and promote the nuclear translocation of ATF4, which increased the transcriptional activity of ILEI. These findings indicated that TMTC3 activates GRP78/PERK signaling pathway during ER stress-induced EMT, which might serve as a potential therapeutic target in SCCs.

## Introduction

Squamous cell carcinomas (SCCs) are a kind of common malignant tumor that mostly originated from epithelial organs, including the skin, esophagus, head and neck, lung, and cervix 1. Although SCCs are sourced from different tissues, they have many similar features, including genomic profiling, histological features, and molecular characteristics 2. Although the technologies for diagnosis and therapy have continuously improved, SCCs are the leading cause of cancer-related mortality and clinical outcome was far from satisfactory. Therefore, it is an urgent task to elucidate the molecular mechanism that contributes to SCCs progression to benefit diagnosis and therapy.

Endoplasmic reticulum (ER) stress is initiated by nutrient deprivation, accumulation of unfolded or misfolded proteins 3, 4. Upon ER stress, the unfolded protein response (UPR) pathway is activated to restore ER homeostasis, which is orchestrated by three major sensors spanning the ER membrane: PERK, ATF6, and IRE1. Under homeostasis conditions, the ER chaperone GRP78, also called binding immunoglobulin protein (Bip), dissociates from ER stress sensors and corrects protein misfolding 5. Additionally, the persistent activation of UPR signaling pathways triggers PERK-ATF4 pathway to endow tumor cells with greater malignant capacity, including proliferation, metastasis, and drug resistance 6. Furthermore, UPR pathways regulated the expression of epithelial-mesenchymal transition (EMT) markers to orchestrate tumor survival and metastasis 7. Accumulating evidence in recent years indicated the tight association between ER stress and EMT, but the detailed mechanism in tumors remains unclear.

Transmembrane and tetratricopeptide repeat (TPR) containing protein 3 (TMTC3) or SMILE, located at ER, is a 914-amino-acid transmembrane protein with 8 transmembrane segments, containing hydrophobic domain at N-terminal and TPR domain at C-terminal. In a cohort of families with recessive forms of cobblestone lissencephaly, which is a severe brain malformation, biallelic mutations in TMTC3 were identified in 6 of 25 families 8. Knockout of TMTC3 was shown to be involved in the O-mannosylation of cadherins by using glycoproteomes 9, 10. Furthermore, TMTC3 was found to regulate O-linked glycosylation and cadherin-mediated adherence, which provides insight into its effect on cellular adherence and migration 11. It was reported that TMTC3 participates in the response to ER stress by interacting with PDIA3 to play a role in immune regulation 12.

The objectives of the present study were to elucidate the connection between ER stress and EMT in tumors. Our findings provide insight into the association of TMTC3 in the regulation of EMT via ER stress and its function in promoting the oncogenic phenotype. Moreover, an SCCs- specific expression pattern was observed for TMTC3, and its promoter was found to interact with ΔNp63, an SCCs-specific transcription factor.

## Materials and Methods

### Cell culture

Esophageal SCC (ESCC) cell lines (except KYSE150) were cultured in RPMI-1640 complemented with 10% fetal bovine serum. The KYSE150 cell line was maintained in RPMI1640 supplemented with Ham's F12 plus 2% fetal bovine serum (RPMI1640: Ham's F12 = 1:1). Ten human ESCC cell lines used in this study were kindly provided by Professor Yutaka Shimada from Kyoto University, Japan. Lung SCC (LUSC) cell line NCI-H520 was cultured in RPMI-1640 complemented with 10% fetal bovine serum. Head and neck SCC (HNSC) cell line FaDu was cultured in DMEM complemented with 10% fetal bovine serum. All cell lines were maintained at 37°C in 5% CO_2_. All cell lines were authenticated by short tandem repeat (STR) profiling.

### Antibodies, siRNA, plasmids and primers

The following antibodies were used in this study: The TMTC3 antibody (Atlas Antibodies Cat# HPA038550, RRID: AB_10672715) for immunohistochemistry (IHC) was purchased from Sigma-Aldrich. The TMTC3 antibodies (ab242111 or sc-398137) for immunoprecipitation (IP) and Western blot were purchased from Abcam and Santa Cruz, respectively. The E-cadherin antibody (Cell Signaling Technology Cat# 3195, RRID: AB_2291471) for Western blot and ATF4 antibody (Cell Signaling Technology Cat# 11815, RRID: AB_2616025), P63 (Cell Signaling Technology Cat# 13109) for Chromatin immunoprecipitation (ChIP) were purchased from Cell Signaling Technology. The N-cadherin antibody ((Abcam Cat# ab18203, RRID: AB_444317) was purchased from Abcam. Vimentin (Proteintech Cat# 10366-1-AP), PERK (Proteintech Cat# 20582-1-AP), IRE1α (Proteintech Cat# 27528-1-AP), ATF6 (Proteintech Cat# 24169-1-AP, RRID: AB_2876891), and ATF4 (Proteintech Cat# 10835-1-AP, RRID: AB_2058600) for Western blot and IF, and GRP78/Bip (Proteintech Cat# 11587-1-AP), β-actin (Proteintech Cat# 66009-1-Ig, RRID: AB_2687938) and ILEI (Proteintech Cat# 14247-1-AP, RRID: AB_2278149) antibodies was purchased from Proteintech.

The siRNAs of TMTC3 were synthesized by GenePharma (Suzhou, China). ATF4, ΔNp63 and ILEI plasmids were synthesized by Genenary (Shanghai, China). The sequences of siRNAs and qPCR primers were provided in Supplementary Table S1.

### Cell proliferation assay and Transwell assay

The experiments were performed as previously described 13. In brief, ESCC cells were seeded into 96-well plates, and the cell proliferation was detected every 15 min by the xCELLigence RTCA-MP (Acea Biosciences/Roche Applied Science, USA). For the Transwell assay, ESCC cells were added to 6.5-mm-diameter chambers with an 8.0-μm pore (Corning, USA). The cells that migrated to the lower chamber were stained with crystal violet and counted.

### Chromatin immunoprecipitation (ChIP) assay

The ChIP procedure was performed according to the instructions of the ChIP analysis kit (ThermoFisher, Shanghai, China). In brief, cells were cross-linked with formaldehyde for 10 min at room temperature, and the reaction was stopped with glycine buffer, and the cells were collected. Then the pellet was resuspended in lysis buffer and sonicated via a sonicator (Covaris) to produce chromatin fragments of approximately 200-500 bp. The sonicated chromatin was incubated with TP63 antibody, or ATF4 antibody or isotype control antibody for overnight at 4°C. Magnetic beads were added to the complex of chromatin and antibody for 3 hours. After the beads were washed, the chromatin bound with the antibody was eluted and DNA was retained after treatment with Proteinase K. Finally, DNA was collected on a column and qPCR was performed to evaluate the enrichment at the TMTC3 promoter or ILEI promoter.

### Dual-luciferase reporter assays

Cells were seeded on 24-well plates. When the confluence reached 50-70%, the cells were co-transfected with TP63 plasmids and TMTC3 promoter plasmids, or ATF4 plasmids and ILEI promoter plasmids. After 48 h of transfection, luciferase activity was measured with the Dual Luciferase Reporter Assay System kit (Promega) and normalized to *R. reniformis* luciferase activity.

### Co-immunoprecipitation assay

ESCC cells were scraped from plates and cell lysates were obtained by applying IP lysis buffer (pH 8.0 Tris-HCl 20 mM, NaCl 137 mM, NP40 1%, EDTA 2 mM). 2 mg of cell lysates was precleared by 2 μg IgG antibody at 4℃ for 1 h and 50 μl Protein A/G beads for 30 min. The supernatant was obtained by centrifugation and incubated with the indicated antibody at 4℃ overnight, and then mixed with 50 μl Protein A/G beads at 4℃ for 6 h. The resulting beads were washed 6 times with IP wash buffer (pH 7.4 Tris-HCl 10 mM, NaCl 150 mM, Triton X-100 1%, EDTA pH 8.0 1 mM) before analysis. The total protein concentration was measured by BCA assay.

### IF staining analysis

ESCC cells were rinsed with PBS and fixed with 4% paraformaldehyde for 10 min. Next, the cells were permeabilized in 0.5% Triton X-100 for 5 min and blocked with normal goat serum for 20 min. Primary antibodies were incubated at 4℃ overnight, and secondary antibodies were applied for 1 h. Finally, the cells were counterstained with DAPI. Images were obtained using a Leica confocal laser scanning microscope.

### Xenograft studies

For subcutaneous tumor growth assessment, the experiments were performed as previously described 13. Briefly, Sh-TMTC3-1 or negative control cells (1×10^6^) in 100 μl were injected subcutaneously into separate flanks of the 4-week-old BALB/C nude mice, which were purchased from Beijing HFK Bioscience Co.,Ltd. Tumor volume was measured every 3 days when the tumors were visible, and calculated using the formula *V* = 0.5 × *W*^2^ × *L* (*V*, volume; *L*, length; *W*, width).

For tail vein metastasis model experiments, Sh-TMTC3-1 or negative control cells (1×10^6^) in 100 μl were injected into the tail vein of the 5-week-old BALB/C nude mice. The injection was given once a week for four times. The mice were sacrificed after 2 months after the first injection, at which time the lungs, livers and lymph nodes were collected and analyzed to determine the rate of metastasis incidence and the number of tumor nodules.

### Statistical analysis

Two-tailed Student's t-test was used for biological experiment analysis. For clinical data, the chi-square test and Fisher's exact test were used for classification variables, and Student's t-test was used for continuous variables. Cumulative survival was calculated by the Kaplan-Meier method, and survival curves were compared by log-rank analysis. A Cox proportional risk model was used to evaluate independent prognostic factors. *p* < 0.05 was considered to indicate statistical significance. All statistical analyses were performed with GraphPad Prism 8 (GraphPad Prism, RRID:SCR_002798) or SPSS (IBM SPSS Statistics, RRID:SCR_016479).

### Ethics Statement

Research involving animals: The procedures complied with international, national, and/or institutional guidelines including welfare and use of animals.

Research involving human patients: All patients signed written informed consent forms and the procedures performed in this study complied with the guidelines of the Ethics Committee of the National Cancer Center/Cancer Hospital Chinese Academy of Medical Sciences.

### Data availability

The public databases used in this study are available in TIMER2.0 (http://timer.cistrome.org/), GEPIA2 (http://gepia2.cancer-pku.cn/#index) and UALCAN databases (http://ualcan.path.uab.edu/). The data generated in this study are available upon request from the corresponding author.

## Results

### Identification a novel ER stress mediator TMTC3 and related to EMT progress

**Figure 1:**
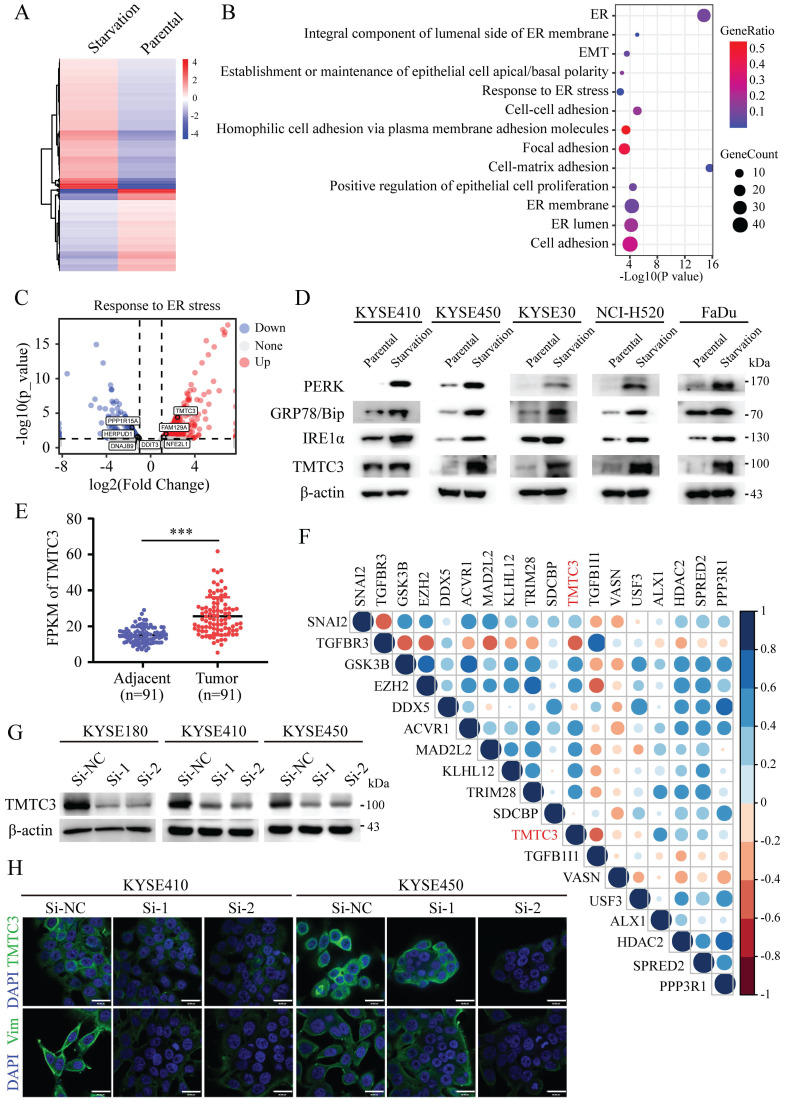
** Identification of TMTC3 in cells under ER stress.** A. Heatmap of DEGs by RNA-seq of human ESCC cell lines treated with complete medium or medium without amino acids for 48 h. B. Pathways associated with ER were altered in cells cultured without amino acid supplementation. C. Volcano plot for genes involved in ER stress, red for upregulated genes and blue for down-regulated genes. D. The protein levels of ER sensors and TMTC3 in SCCs cells, including ESCC, LUSC, HNSC. E. The FPKM of TMTC3 in transcriptome sequencing data in 91 paired ESCC tumors and adjacent normal tissues. F. Cross correlation analysis of reads between TMTC3 and EMT markers from transcriptome sequencing data in 91 paired ESCC tissues. G. The knockdown efficiency of TMTC3 by siRNA in KYSE180, KYSE410 and KYSE450 cells detected by Western blot. H. Representative IF staining images showing the expression of TMTC3 and Vimentin in KYSE410 and KYSE450 cells. The scale bar represents 30 μm.

To understand the underlying mechanism of ER stress inducing EMT, KYSE450 cells were treated with complete medium or with amino acid and FBS-deficient medium for 24 h to induce ER stress. The mRNA expression of ER stress sensors, including *HSPA5*, *EIF2AK3*, *ERN1*, *ATF6*, and etc., was upregulated in KYSE450 cells cultured by amino acid and FBS-deficient medium, which indicated the ER stress model was successfully established (Supplementary Fig. S1A). To identify potential modulators of ER stress, we analyzed the gene expression profiles of ESCC cell line in the context of ER stress. The differentially expressed genes (DEGs) were screened according to the criteria of log_2_|foldchange|≥ 1 and *p* ≤ 0.05, which revealed 446 upregulated genes and 281 downregulated genes (Fig. 1A). Pathway enrichment based on the DEGs was performed by online database KOBAS3.0 (http://kobas.cbi.pku.edu.cn/kobas3), among which ER stress signaling pathway was significantly altered (Fig. 1B). Moreover, pathways associated with cell adhesion and EMT were enriched and obviously changed, indicating the correlation between ER stress and EMT (Fig. 1B). TMTC3 was identified and obviously upregulated among the screened genes in the response to ER stress pathway (Fig. 1C). Subsequently, the protein levels of TMTC3, GRP78/Bip, PERK, IRE1α were increased in cells treated with amino acids and FBS free medium compared with cells treated with complete medium (Fig. 1D). Taken together, these findings indicated that the expression of TMTC3 is elevated in SCCs cells undergoing ER stress.

In order to explore the relationship between TMTC3 and EMT, the expression of TMTC3 was analyzed in a set of 91-paired ESCC tissues with transcriptome sequencing data, and the results indicated obvious upregulation in tumor tissues compared with adjacent tumor tissues (Fig. 1E). Furthermore, the correlation results indicated TMTC3 was significantly associated with several genes that regulated EMT progress (Fig. 1F). Additionally, we detected the protein expression of TMTC3 was analyzed in ESCC cell lines (Supplementary Fig. S1B). KYSE180 cells with high TMTC3 expression and KYSE410 and KYSE450 cells with moderate TMTC3 expression were chosen for further experiments. The knockdown efficiency of TMTC3 by siRNAs was measured by western blot and qPCR analysis (Fig. 1G and Supplementary Fig. S1C). Immunofluorescence staining suggested lower Vimentin protein expression in TMTC3 knockdown cells than in control cells (Fig. 1H). Taken together, TMTC3 was involved in the process of EMT in ESCC cells.

### Ectopic expression of TMTC3 in SCCs

To systematically evaluate the mRNA level of TMTC3 in various cancers, we performed an in silico analysis using the Tumor IMmune Estimation Resource (TIMER) 2.0 database to analyze the mRNA expression of TMTC3 in human normal tissues and cancerous tissues. We observed a significant elevation of its expression in a series of tumors (Supplementary Fig. S1D). Moreover, in the UALCAN database, we found that the expression of TMTC3 in ESCC was higher than that in the other esophageal cancer subtype, esophageal adenocarcinoma (EAC) (Supplementary Fig. S1E). Additionally, the expression of TMTC3 in other SCCs, including head and neck SCC (HNSC), lung SCC (LUSC), was significantly upregulated than adenocarcinoma (Supplementary Fig. S1E). Moreover, the expression of TMTC3 was detected in HNSC and LUSC cells undergoing ER stress and the results indicated that the TMTC3 and all ER stress sensors levels were increased (Fig. 1D). Thus, the expression of TMTC3 is upregulated in various tumors, especially in SCCs.

**Figure 2:**
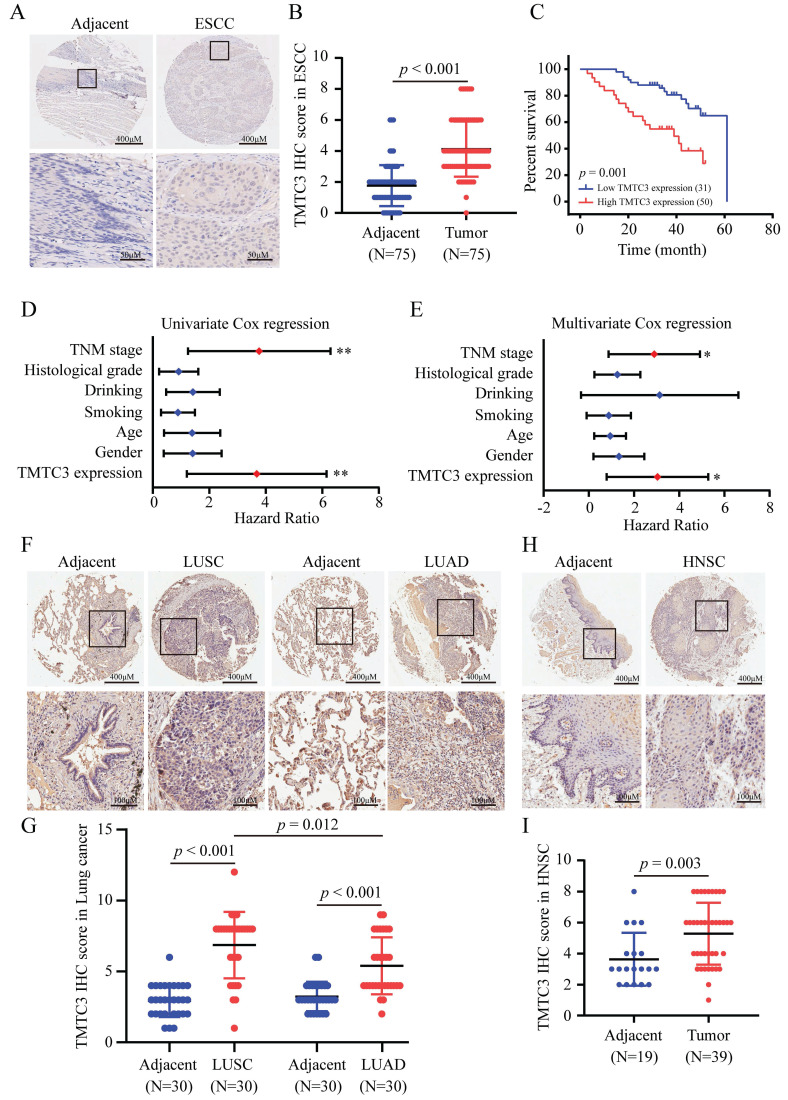
** Ectopic expression of TMTC3 in SCCs tissues.** A. Representative IHC images of TMTC3 in the ESCC tissue array. B. IHC staining score for each ESCC tissue spot was calculated based on the intensity and area of the staining for TMTC3. C. High expression of TMTC3 was associated with poor survival in ESCC patients. D and E. Univariate (D) and multivariate (E) Cox regression survival analysis of correlations between overall survival and other clinicopathological features. *, *p* < 0.05. **, *p* < 0.01. F and G. Representative IHC images (F) and IHC staining score for TMTC3 (G) in lung cancer tissue array. H and I. Representative IHC images (H) and IHC staining score for TMTC3 (I) in HNSC tissue array.

To investigate the expression of TMTC3 in SCCs, an IHC assay for evaluating the protein level of TMTC3 was performed via tissue microarrays. Obviously, the expression level of TMTC3 in ESCC tissues was significantly upregulated compared with that in paired adjacent peritumoral tissues (Fig. 2A and 2B). We further explored the correlation between TMTC3 expression and the clinical characteristics in transcriptome sequencing based on 91-paired ESCC tissues. Statistical analysis revealed that TMTC3 expression was positively associated with TNM stage in ESCC patients (*p* = 0.05) (Supplementary Table S2). Kaplan-Meier and univariate Cox regression analyses revealed that high expression of TMTC3 was strongly associated with poor survival (median survival, 39 months *vs.* 61 months, *p* = 0.001) (Fig. 2C and 2D). Univariate Cox regression analysis also indicated that TNM stage III was a poor prognostic factor in terms of overall survival (Fig. 2D and Supplementary Table S2). Moreover, subsequent multivariate analysis with the Cox proportional hazards model revealed that both TMTC3 expression and TNM stage were prognostic factors for the prediction of overall survival (Fig. 2E and Supplementary Table S2). In addition, the expression of TMTC3 in other SCCs was evaluated by IHC staining. As shown in Fig. 2F and 2G, it exhibited higher expression of TMTC3 in LUSC than LUAD, consistent with the mRNA level (Supplementary Fig. S1D and S1E). Meanwhile, in HNSC, the similar result was observed (Fig. 2H and 2I). Taken together, these findings suggest TMTC3 is upregulated and associated with poor survival in SCCs.

### The activation of TMTC3 was regulated by the SCCs-specific transcription factor ΔNp63

TMTC3 was upregulated in various cancers, especially in ESCC, LUSC, HNSC. However, TMTC3 was not located in the chromosomal amplified region according to our previous research in ESCC 14. Thus, we sought for the transcription factors that could bind the promoter of TMTC3 to stimulate the process of transcription and are also specifically expressed in SCCs.

**Figure 3:**
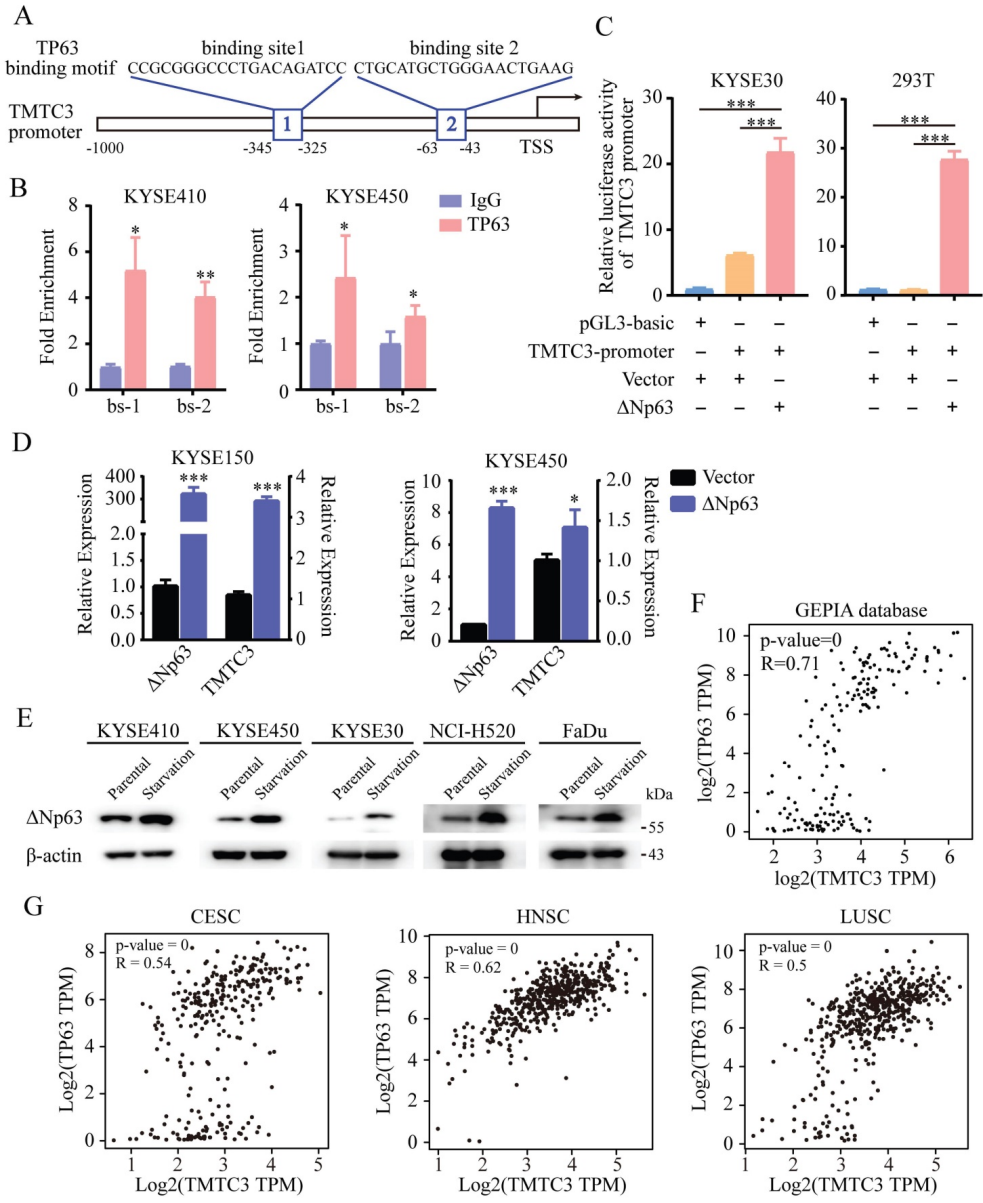
** TMTC3 was regulated by ΔNp63 at the transcriptional level.** A. Schematic diagram depicting the positions of the binding sites of TP63 in the TMTC3 promoter from the JASPAR database. TSS, transcription starting site. B. ChIP-qPCR analysis of the interaction between ΔNp63 and the TMTC3 promoter. IgG was used as a negative control. *, *p* < 0.05, **, *p* < 0.01. C. The luciferase activity of TMTC3 after stimulation with ΔNp63. ***, *p* < 0.001. D. The mRNA expression of TMTC3 after overexpressing ΔNp63. E. The ΔNp63 levels in SCCs cells treated with complete medium or with amino acid and FBS-deficient medium for 24 h. F and G. The correlation between TP63 and TMTC3 in EC (F) and other SCCs, including CESC, HNSC, LUSC (G) from GEPIA2 database.

Previous studies have demonstrated that the N-terminally truncated isoform of transcription factor p63 (ΔNp63), the predominant isoform in SCCs, is a master regulator of initiation and progression in SCCs 15-17. Thus, we explored whether TP63 is the regulator of TMTC3. Indeed, analysis of the ChIP-seq database indicated a conserved putative TP63 binding element in the TMTC3 promoter (Fig. 3A). To further explore whether TMTC3 was directly regulated by TP63, ChIP-qPCR analysis was performed to confirm the binding relationship between the TP63 protein and the TMTC3 promoter. The results showed that TP63 but not the IgG control could bind the predicted sites (Fig. 3B). To further investigate the interaction of ΔNp63 and the TMTC3 promoter, we conducted a dual-luciferase reporter assay. The results showed that the luciferase activity after co-transfection of the ΔNp63 plasmid and TMTC3 promoter plasmid was obviously increased compared with that of the negative control group (Fig. 3C). In addition, overexpression of ΔNp63 enhanced the mRNA level of TMTC3 in ESCC cells (Fig. 3D). Additionally, it was reported that TP63 has been implicated as a mediator of stress signals, including ER stress and oxidative stress 18. The expression of ΔNp63 was activated by ER stress in zebrafish 19. We also verified that the expression of TP63 was upregulated in ER stress cells, including ESCC, LUSC and HNSC (Fig. 3E). Moreover, a strong positive correlation between TMTC3 and TP63 was confirmed in esophageal cancer (EC) (Fig. 3F). The same results in CESC, HNSC and LUSC were obtained from GEPIA2 database (Fig. 3G). Together, these findings indicated the expression of TMTC3 was regulated by TP63, an SCCs-specific transcription factor.

### Knockdown of TMTC3 inhibited the malignant phenotype *in vitro*

**Figure 4:**
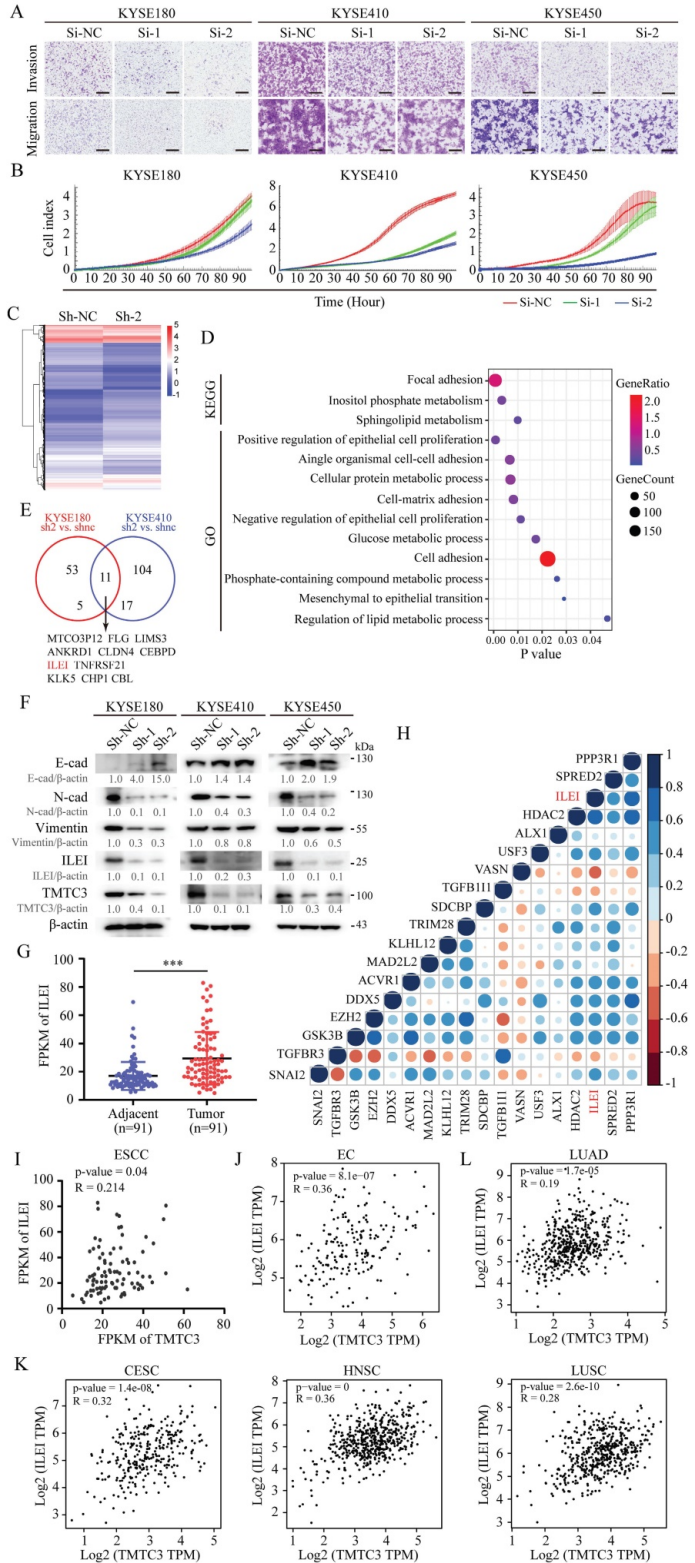
** TMTC3 promoted EMT progress via ILEI.** A. The representative images of the Transwell assay in ESCC cells transfected with siRNA for TMTC3. The scale bar represents 500 μm. B. Effects of TMTC3 siRNA on ESCC cell growth via RTCA assays. C. Heatmap of DEGs by RNA-seq in KYSE180 cells stably infected with Sh-TMTC3 or Sh-NC. D. The significantly enriched KEGG pathways and GO terms for DEGs from RNA-seq in TMTC3-knockdown KYSE180 cells. E. Venn diagram of TMTC3 downstream genes from two RNA-seq analysis and 11 genes were selected. F. Western blot analysis for E-cadherin (epithelial marker), N-cadherin and Vimentin (mesenchymal marker) and ILEI in Sh-TMTC3 cells. G. The FPKM of ILEI in transcriptome sequencing data in 91 paired ESCC tumors and adjacent normal tissues. H. Cross correlation analysis of reads between ILEI and EMT markers from transcriptome sequencing data in 91 paired ESCC tissues. I. The positive correlation between TMTC3 and ILEI from transcriptome sequencing data in 91 paired ESCC tissues. J and K. The Pearson's correlation coefficient between TMTC3 and ILEI in EC (J) and other SCCs, including CESC, HNSC, LUSC (K) from GEPIA2 database. L. The correlation between TMTC3 and ILEI in LUAD.

Then, we explored whether TMTC3 could influence the ESCC cell malignant phenotype*.* As shown in Fig. 4A and Supplementary Fig. S2A, knockdown of TMTC3 by siRNA obviously inhibited migration and invasion of ESCC cell lines. Furthermore, Real-Time Cell Analyzer (RTCA) assays showed that KYSE180, KYSE410 and KYSE450 cells transfected with TMTC3 siRNA exhibited decreased cell proliferation (Fig. 4B). To extend our findings, three stable cell strains (in KYSE180, KYSE410 and KYSE450 cells) with TMTC3 knockdown were established via two shRNA lentiviral vectors and a nontargeting vector. In addition, Western blot analysis confirmed the knockdown efficiency at the protein level, and qPCR experiments were applied to evaluate the mRNA expression of TMTC3 (Supplementary Fig. S2B and S2C). Similarly, stable TMTC3 downregulation significantly attenuated the malignant phenotype in ESCC cells, as indicated by reduced migration, invasion and proliferation (Supplementary Fig. S2D-F). In summary, TMTC3 functions as an oncogene to promote ESCC progression.

### The reduction of TMTC3 inhibited EMT progress through ILEI

To further investigate the mechanisms underlying TMTC3-mediated cellular effects, RNA-seq analysis was performed on cells with stable TMTC3 knockdown. The heatmap plots of DEGs from RNA-seq analysis (KYSE180 Sh-2 *vs.* KYSE180 Sh-NC, KYSE410 Sh-2 *vs.* KYSE410 Sh-NC, |fold change| ≥ 2) were shown in Fig. 4C and Supplementary Fig. S3A. Moreover, pathway analyses showed that TMTC3 expression level was closely associated with cell adhesion in ESCC (Fig. 4D and Supplementary Fig. S3B). Subsequently, we analyzed the genes that upregulated in both RNA-seq analyses, and 11 genes were screened, among which the expression of ILEI was higher in ESCC than in EAC via the UALCAN database (Fig. 4E and Supplementary Fig. S3C). It has been reported that ILEI is associated with EMT progress in human breast cancer and colon cancer 20-22. Thus, EMT markers and ILEI were detected in TMTC3 cells with stable knockdown, and the results showed that E-cadherin was upregulated, N-cadherin, Vimentin and ILEI was downregulated in TMTC3 knockdown cells compared with control cells (Fig. 4F). The mRNA levels of both ILEI and Vimentin were reduced in Sh-TMTC3 cells (Supplementary Fig. S3D). The expression of ILEI in 91-paired ESCC tissues was analyzed and correlation analysis between ILEI and EMT-associated genes was performed. The results indicated that the upregulated expression of ILEI was positively correlated with EMT-associated genes (Fig. 4G and 4H). Considering of ILEI as the downstream of TMTC3, the level of ILEI in SCCs cells in the context of ER stress was detected, which indicated the obviously upregulation in comparison with parental cells (Supplementary Fig. S3E). Additionally, the positive correlation between TMTC3 and ILEI was observed in a set of 91-paired ESCC tissues and GEPIA2 database, respectively (Fig. 4I and 4J). Additionally, a strongly positive correlation between TMTC3 and ILEI was identified in CESC, HNSC and LUSC from GEPIA2 database (Fig. 4K). In contrast, the comparable significant correlation was not observed in LUAD (Fig. 4L). These results suggested that TMTC3 might affect EMT progress through ILEI, in an SCCs-specific pattern.

### TMTC3 disrupted the interaction between GRP78 and PERK

**Figure 5:**
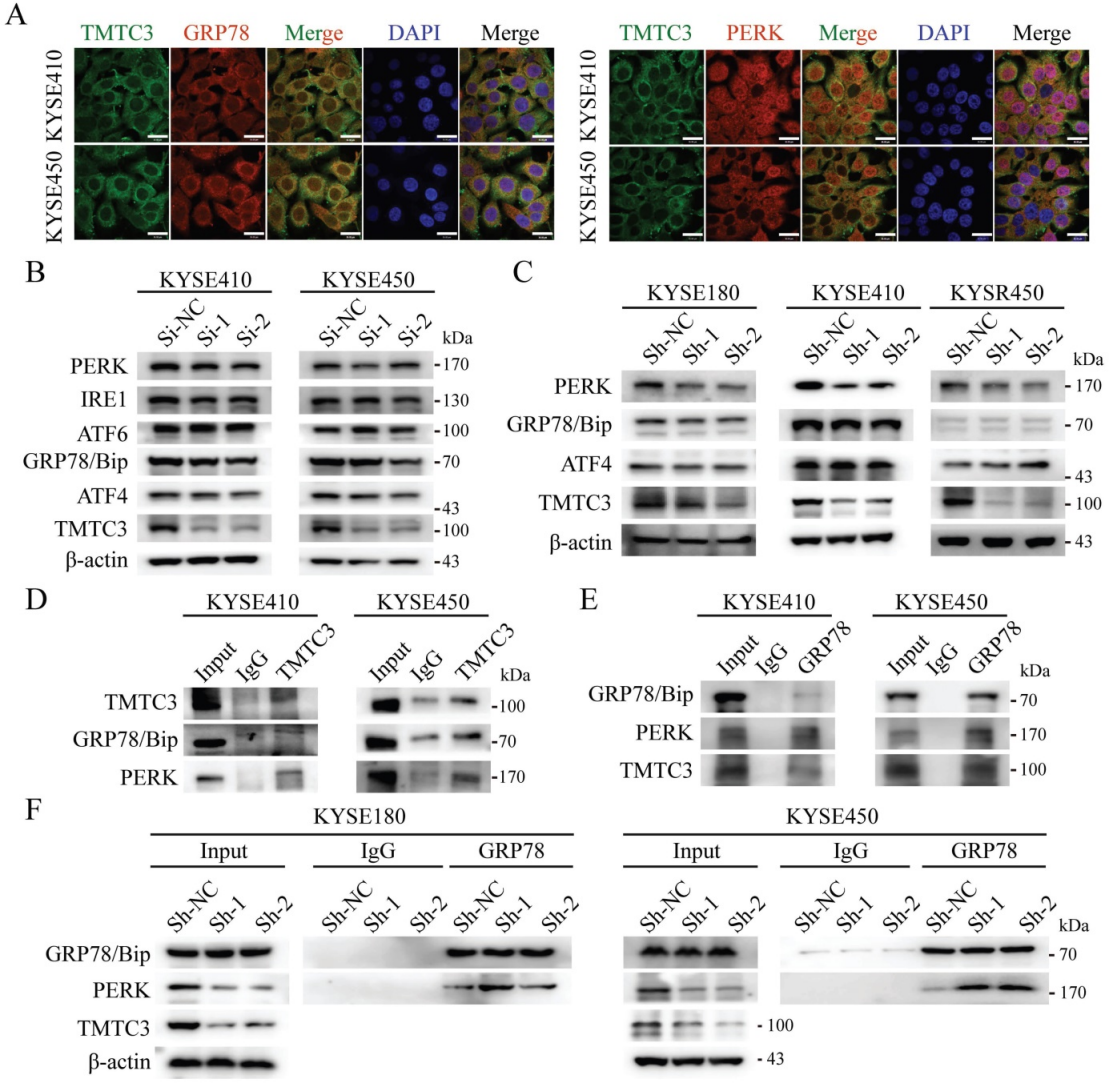
** TMTC3 enhanced ER stress by disturbing the interaction between GRP78 and PERK.** A. Colocalization of TMTC3 and GRP78 or PERK in cell lines by IF. Scale bar, 30 µm. B and C. Expression of ER sensors in ESCC negative control and Si-TMTC3 (B) or Sh-TMTC3 (C) cells. D and E. Co-immunoprecipitation of TMTC3, GRP78 and PERK in cell lines. F. Co-immunoprecipitation and Western blot analysis for GRP78 and PERK in Sh-TMTC3 or Sh-NC cells.

According to the previous researches, TPR domain of TMTC3 mediated protein-protein interaction, not responsible for RNA binding. As TMTC3 is located at the ER membrane, we proposed the function of TMTC3 depending on its cellular location. It has been reported that ER stress effectors may promote EMT, as an additional mechanism for EMT activation 7. Therefore, we explored whether TMTC3 mediated EMT progress via ER stress. Firstly, IF staining of TMTC3 and GRP78/Bip, PERK, ATF6, and IRE1α was conducted. The results confirmed the colocalization of TMTC3 and ER-associated proteins in ESCC cell lines (Fig. 5A and Supplementary Fig. S4).

Subsequently, the expression of GRP78/Bip and PERK, IRE1α and ATF6 were detected in siRNA or shRNA-treated ESCC cells, and the results showed a reduction in PERK in TMTC3 knockdown cells (Fig. 5B and 5C). Although the expression of GRP78/Bip was not affected by TMTC3, GRP78/Bip was released from protein complex to activate the UPR pathway. Therefore, we hypothesized that TMTC3 suppresses the association between GRP78/Bip and PERK by interacting with GRP78/Bip. A co-immunoprecipitation assay was conducted to analyze the interaction among GRP78/Bip, PERK and TMTC3 via GRP78/Bip or TMTC3 antibodies. The results demonstrated that GRP78/Bip interacted with TMTC3 and PERK in KYSE410 and KYSE450 cells (Fig. 5D and 5E). As shown in Fig. 5F, GRP78/Bip and PERK interaction signals were obviously increased in TMTC3 knockdown cells. These results indicate that TMTC3 activates ER stress via partial suppression of the GRP78/Bip and PERK interaction.

### TMTC3 knockdown suppressed the transcription of ILEI via ATF4

**Figure 6:**
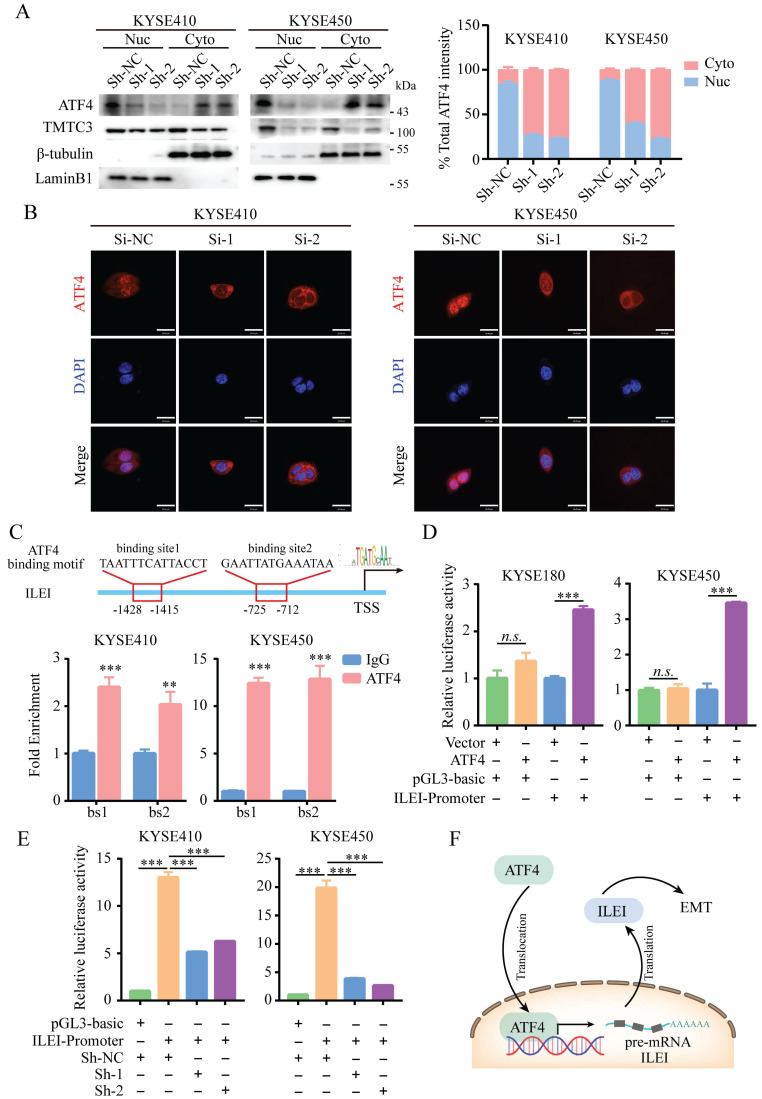
** ATF4 increased the transcriptional activity of ILEI to promote EMT in ESCC.** A. ATF4 protein expression in fractionated cells with stably expressed Sh-TMTC3 and Sh-NC (left). The quantification of ATF4 intensity in WB (right). Nuc, nuclear. Cyto, cytoplasm. β-tubulin is a cytoplasmic control, LaminB1 is a nuclear control. B. IF images for the localization of ATF4 in TMTC3 cells transfected with siRNA and negative control. Scale bar, 30 µm. C. Binding of ATF4 to the ILEI promoter region in KYSE410 and KYSE450 cells was analyzed by ChIP-qPCR. IgG was used as a nonspecific binding control. **, *p* < 0.01. ***, *p* < 0.001. D. The relative luciferase reporter activity of ATF4 on the ILEI promoter in KYSE180 and KYSE450 cells. ***, *p* < 0.001. *n.s.*, no significance. E. The relative dual-luciferase activity of the ILEI promoter in TMTC3 cells with stable knockdown. ***, *p* < 0.001. F. Model of the ATF4-ILEI axis in regulating EMT in SCCs.

A previous study suggested that TMTC3 is involved in ER stress and activates the PERK pathway but does not obviously affect the total protein levels of ATF4, the downstream effector of PERK (Fig. 5B and 5C). We next investigated whether TMTC3 affected the nuclear translocation of ATF4, a transcription factor essential for ER stress. Western blot analysis of the nucleoplasm fractionation assay indicated that knockdown of TMTC3 decreased the accumulation of ATF4 in the nucleus (Fig. 6A). To further confirm this observation, we performed IF to examine the effect of TMTC3 on ATF4 expression and localization. Here, ATF4 staining in the nucleus was decreased in cells with stable TMTC3 down-regulation (Fig. 6B). The previous researches showed that the expression of ATF4 was strongly associated with EMT signatures expression. Since ATF4 is a transcription factor, we investigated whether ATF4 affects the transcription of ILEI, the downstream gene of TMTC3.

According to the prediction of the JASPAR database, two putative ATF4 binding sites (bs1, bs2) in the ILEI promoter were found, and corresponding primers were designed around the binding sites. The ChIP-qPCR results showed that ATF4 was highly enriched in the ILEI promoter in KYSE410 and KYSE450 cells (Fig. 6C). Subsequently, a dual-luciferase reporter assay was performed, which indicated that ATF4 overexpression could significantly increase the luciferase activity of the ILEI promoter (Supplementary Fig. S5A-B and Fig. 6D). Next, to further investigate whether TMTC3 could affect the transcription of ILEI, we detected the luciferase activity of the ILEI promoter in TMTC3 knockdown cells and control cells through a dual-luciferase reporter assay. The results suggested that decreased expression of TMTC3 inhibited ILEI promoter activity via the reduced localization of ATF4 (Fig. 6E). Furthermore, a positive correlation between ATF4 and ILEI was observed in ESCC from the TIMER2.0 and GEPIA2 databases (Supplementary Fig. S5C and S5D). Meanwhile, the correlation in other SCCs was analyzed and similar results were obtained (Supplementary Fig. S5E). Therefore, ATF4, as a transcription factor involved in ER stress, could increase the transcriptional activity of ILEI, the downstream gene of TMTC3, to promote EMT progression (Fig. 6F).

### TMTC3 silencing decreased tumor metastasis and growth* in vivo*

**Figure 7:**
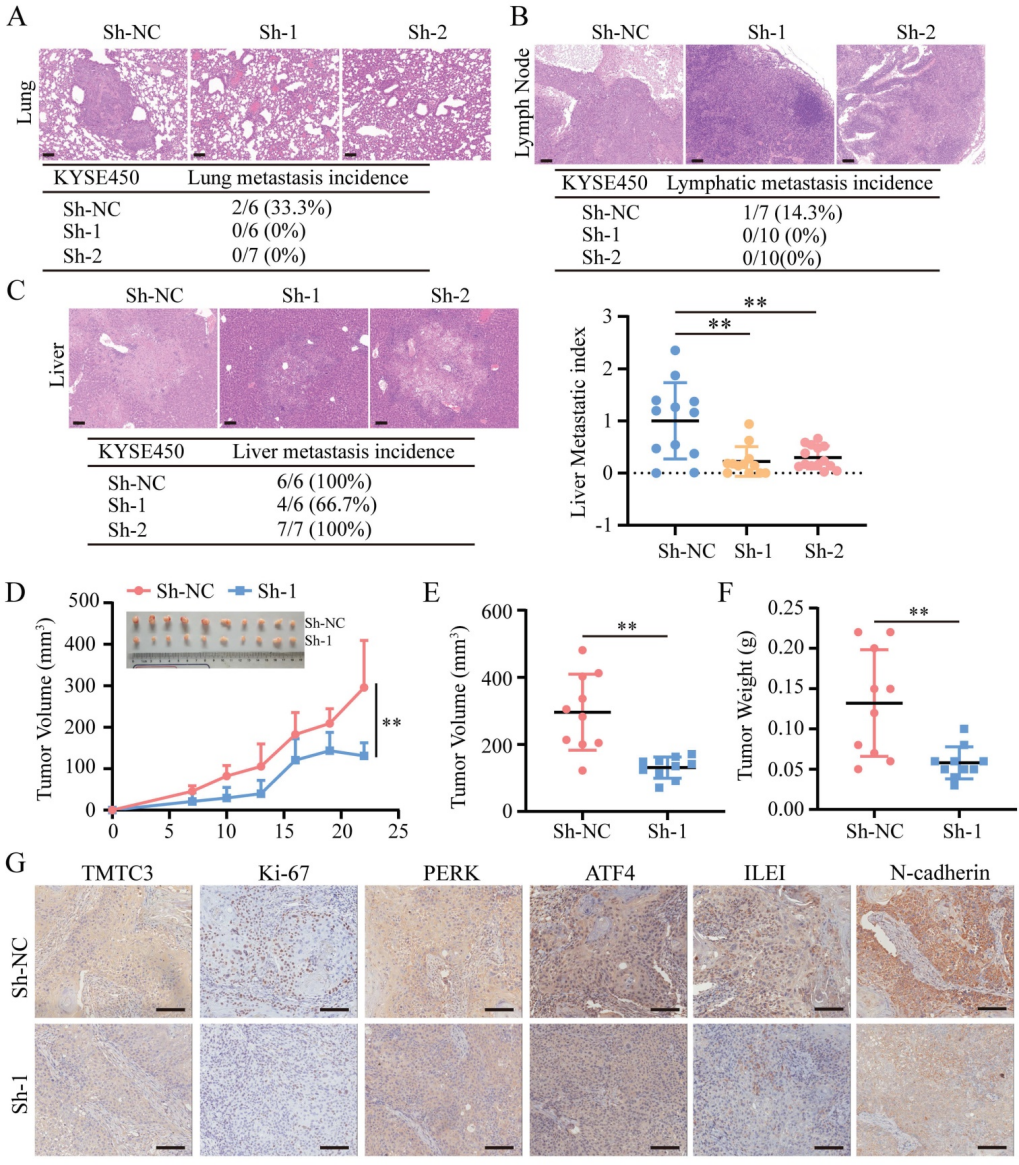
** TMTC3 silencing inhibited tumor metastasis and growth *in vivo*.** A-C. Hematoxylin and eosin (H&E) staining of metastasis to the lung (A), lymph node (B) and liver (C). The scale bar represents 100 μm. The incidence of metastasis including lung, lymph node and liver metastasis, after tail injection. Additionally, the metastatic nodule numbers in the liver were counted. D. Curve analysis of xenograft tumor growth for bearing lenti-Sh-TMTC3 cells and negative control cells. **, *p* < 0.01. E. The volume of xenograft tumors after mice were sacrificed. **, *p* < 0.01. F. The xenograft tumor weight of lenti-Sh-TMTC3 cells compared with Sh-NC cells. **, *p* < 0.01. G. The IHC staining of xenograft tumor for TMTC3, Ki-67, PERK, ATF4, ILEI and N-cadherin. Scale bar, 100 μm.

To further clarify whether TMTC3 could promote cell metastasis and growth *in vivo*, 2 pooled sets of KYSE450 cells that were stably infected with negative control or Sh-TMTC3 lentiviral vectors, were injected into the tail veins of BALB/C nude mice. The mice were sacrificed after two months, and the metastases were analyzed. TMTC3 silencing reduced the incidence of lung metastases (Fig. 7A). Additionally, the effect of TMTC3 on lymphatic metastasis was investigated, and the results showed that the ratios of lymphatic metastases were obviously higher in the negative control group than in the Sh-TMTC3 group (Fig. 7B). Strikingly, metastatic nodules on the liver were visible in all groups. Therefore, the numbers and sizes of liver nodules were counted in each tissue. The metastatic index in sh-TMTC3 groups was lower than that in the negative control group (Fig. 7C). For the subcutaneous xenograft model, the volumes of tumors were recorded before the mice were sacrificed and plotted as a curve (Fig. 7D). In agreement with the *in vitro* results, both tumor volume and weight were obviously reduced in the Sh-TMTC3 group compared with the control group (Fig. 7E and 7F). IHC staining analysis showed that the expression levels of TMTC3 and Ki67, PERK, ATF4, ILEI, and N-cadherin were all reduced in Sh-TMTC3 tumors (Fig. 7G). In summary, downregulation of TMTC3 decreased the malignancy phenotype *in vivo*.

## Discussion

In this study, we identified that TMTC3 as a novel ER stress mediator promoted EMT progress through disrupting the interaction between GRP78 and PERK in ESCC cell lines. In addition, the expression of TMTC3 exhibited an SCCs-specific expression pattern, regulated by SCCs-specific transcription factor TP63.

ER stress phenomenon was triggered by the disorder of many factors in the tumor microenvironment, including ischemia, hypoxia, and nutritional deprivation 23. Under ER stress conditions, PERK signaling pathway was activated, which led to the activation of invasion-metastasis cascade and further induced EMT progress 24. Several studies have reported that ER stress was involved in ESCC progression 25, 26, but the detailed molecular mechanism is still unclear. In this study, we found that TMTC3 is upregulated in SCCs cells undergoing ER stress and associated with EMT behavior. We performed an extensive analysis of the TIMER2.0 database and found TMTC3 is overexpressed in various tumors, especially SCCs. Furthermore, TMTC3 was ectopically expressed in SCCs tissue arrays and enhanced the malignant phenotype *in vitro* and tumor growth and metastasis *in vivo*. RNA-seq analysis in TMTC3 knockdown cells showed that TMTC3 upregulated ILEI mRNA level. Previous studies have shown that ILEI is an important secreted factor 21. It has been reported that ILEI plays a critical role in promoting EMT and metastasis in various cancers, including breast cancer 27, gastric cancer 28, melanoma 29, colorectal cancer 30, and liver cancer 31, either independently or in combination with TGF-β. In addition, ILEI, associated with aggressive tumor behavior, metastasis, and poor clinical outcome, might be a valuable biomarker for the prediction of ESCC prognosis 32. In this study, our data demonstrated that TMTC3 knockdown inhibited EMT progress. Additionally, ILEI level was upregulated in SCCs cells undergoing ER stress, indicating the tight association of ILEI and ER stress.

**Figure 8:**
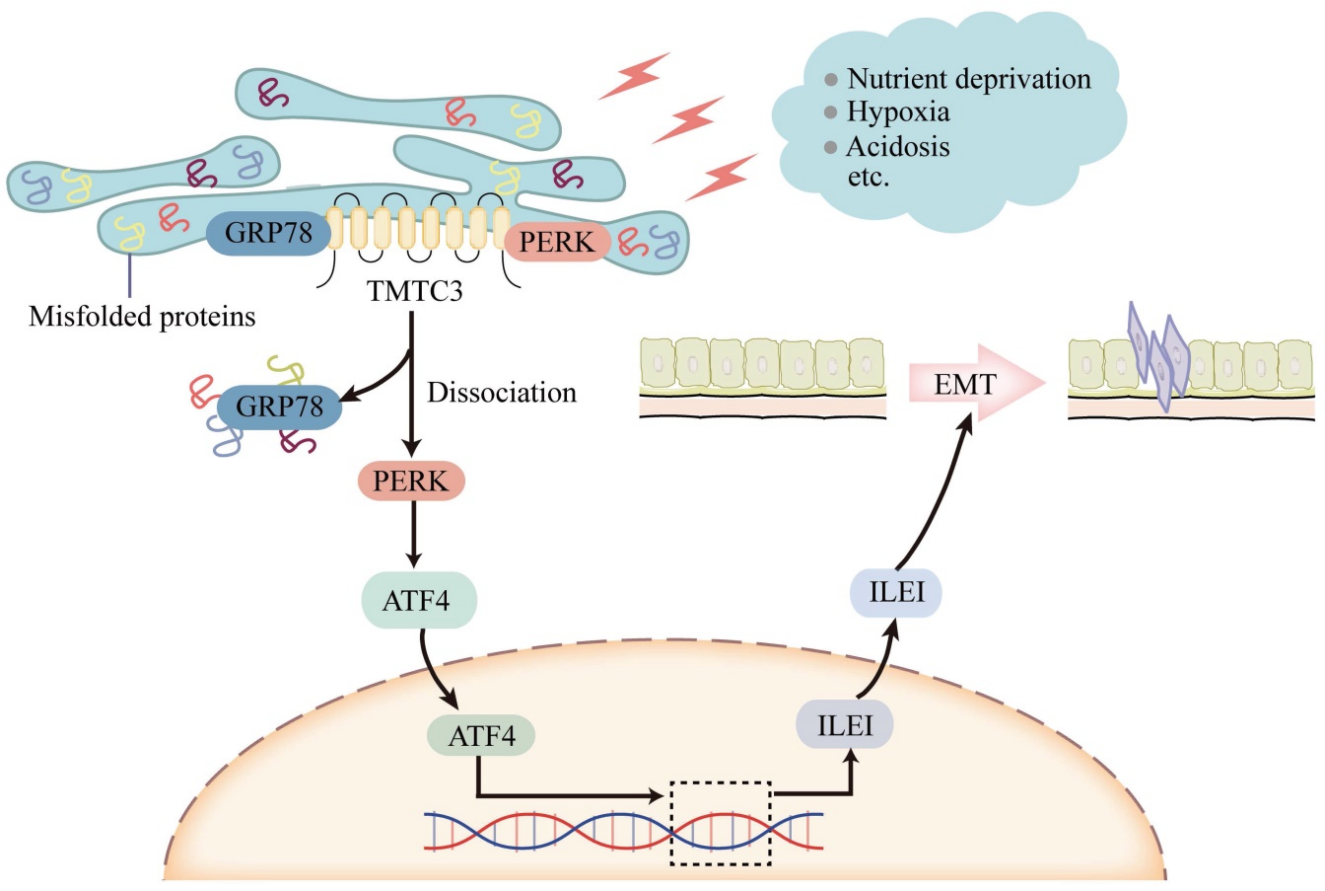
Model of TMTC3 regulating SCCs metastasis via the PERK/ATF4/ILEI axis.

Although TMTC3 upregulated the mRNA level of ILEI, it is not an RNA-binding protein. Additionally, the C-terminal of TMTC3 is mainly composed of tetratricopeptide repeat (TPR) motifs, which mediates protein-protein interaction 11. In consideration of the cellular location of TMTC3, we assessed the interaction between GRP78 and PERK in ESCC cells. Interestingly, high expression of TMTC3 led to the disassociation of PERK from GRP78 and enhanced the activation of PERK/ATF4 in ESCC (Fig. 8). The nuclear location of ATF4 supports cell survival and metastasis via the reconstruction of protein homeostasis 33. It has been reported that glutamine starvation induces EMT progress through upregulation of Slug dependent on ATF4 34. Here, we clarified ATF4, as a transcription factor, enhanced the transcriptional activity of ILEI to promote EMT progress, which was abrogated by knockdown TMTC3.

TMTC3 exhibits a tissue-specific expression pattern, and is overexpressed in SCCs. As demonstrated previously, SCCs originating from different organs have similar phenotypic and molecular characteristics, and are affected by several oncogenes and tumor suppressors 1, 14, 35. ΔNp63 is the predominant isoform of TP63, an amplified oncogene in SCCs 36. Genome-wide approaches have demonstrated the significant role of TP63 in regulating keratinocyte proliferation and differentiation and promoting gene transcription by binding to their promoters or enhancers in a cell-type-specific manner 37, 38. Otherwise, several researches reported that p63 transcription factor was involved in ER stress 18, 19. In this study, we verified that the expression of ΔNp63 was obviously upregulated in SCCs cells in the context of ER stress. Bioinformatics predictions methods indicated that TP63 acted as an upstream regulator of TMTC3 by directly binding to its promoter. ChIP-qPCR assays and dual-luciferase reporter assays further indicated that the upregulation of TMTC3 transcription was regulated by TP63 via potential binding sites. The mRNA level of TMTC3 was increased in ΔNp63 overexpressing cells compared to control cells.

An increasing body of work has that indicated targeting molecules involved in ER stress could inhibit tumor malignancy and enhance the sensitivity of cancer cells to cytotoxic drugs, targeted therapy and immunotherapy. We identified TMTC3 as a novel ER stress response mediator triggered by nutrient in SCCs. Therefore, targeting TMTC3, may be a potential strategy for the clinical therapy of SCCs.

## Supplementary Material

Supplementary figures and tables.

## Abbreviations

TMTC3: Transmembrane O-manosyltransferase targeting cadherins 3
ER stress: Endoplasmic reticulum stress
EMT: epithelial-mesenchymal transition
UPR: Unfolded protein response
SCCs: squamous cell carcinomas
ESCC: Esophageal SCC
EAC: Esophageal adenocarcinoma
HNSC: Head and neck SCC
LUSC: Lung SCC
LUAD: Lung adenocarcinoma
CESC: Cervical SCC
ILEI: interleukin-like EMT inducer
Bip: binding immunoglobulin protein
ChIP: chromatin immunoprecipitation
HSPA5: Heat shock protein family A member 5
GRP78: Glucose-Regulated Protein, 78kDa
EIF2AK3: Eukaryotic translation initiation factor 2 alpha kinase 3
PERK: PRKR-Like Endoplasmic Reticulum Kinase
ERN1: Endoplasmic reticulum to nucleus signaling 1
IRE1α: Inositol-requiring enzyme 1 alpha
ATF6: Activating transcription factor 6
ATF4: Activating transcription factor 4
DEGs: differentially expressed genes
TIMER: Tumor IMmune Estimation Resource
ΔNp63: N-terminally truncated isoform of transcription factor p63
RTCA: Real-Time Cell Analyzer
qPCR: quantitative real-time PCR
