# Supplementary material for: A Novel ER Stress Mediator TMTC3 Promotes Squamous Cell Carcinoma Progression by Activating GRP78/PERK Signaling Pathway
Source: Int J Biol Sci. 2022 Jul 18;18(13):4853–68. doi: 10.7150/ijbs.72838 (PMC9379397; doi:10.7150/ijbs.72838)
Supplement: Supplementary file 1 — Supplementary figures and tables. [file ijbsv18p4853s1.pdf]

A novel ER stress mediator TMTC3 promotes squamous cell carcinoma progression by activating GRP78/PERK signaling pathway

Hongyu Yuan[1,†], Zitong Zhao[1,†], Zichan Guo[1], Liying Ma[1], Jing Han[2], Yongmei Song[1,*]

Affiliations:

[1]State Key Laboratory of Molecular Oncology, National Cancer Center/National Clinical Research Center for Cancer/Cancer Hospital, Chinese Academy of Medical Sciences and Peking Union Medical College, Beijing 100021, China.

[2]Department of Medical Oncology, Hebei Medical University Fourth Affiliated Hospital and Hebei Provincial Tumor Hospital, Shijiazhuang, Hebei 050000, China.

[*]Corresponding author. Tel: +86 10 8778 7498, fax: +86 10 6771 5058. E-mail addresses: symlh2006@163.com, songym@cicams.ac.cn.

[†]These authors contributed equally to this work.

Supplementary Table Legends

Supplementary table 1 The siRNA sequences for TMTC3 and primers for qPCR.

The siRNA sequences for TMTC3.

Gene	Sense (5'-3')	Antisense (5'-3')
TMTC3		
Si-1	GCAUCCAUCUACACCUUUATT	UAAAGGUGUAGAUGGAUGCTT
Si-2	GCGCUUUGUUUAAUGGCAUTT	AUGCCAUUAAACAAAGCGCTT

Primer sets for qPCR analysis.

Gene	Forward	Reverse
TMTC3	TGGTTACTGCCTGCTATTGGA	GTGGCTTCTCTCCTCAGACA
β-actin	CTCCATCCTGGCCTCGCTGT	GCTGTCACCTTCACCGTTCC
ILEI	CGGAGGAGTCCGAGAGGAA	CTTTGCAGCACCTGCTACCC
HSPA5	CTTGCCGTTCAAGGTGGTTG	TCTTTGGTTGCTTGGCGTTG
EIF2AK3	AGCCAATTCAATGCCTGGGA	ACTTCTCTGGTGGTGCTTCG
ATF4	AGGAGGAAGACACCCCTTCA	ATCGTAAGGTTTGGGACGGG
ATF6	CCCGTATTCTTCAGGGTGCT	TCACTCCCTGAGTTCCTGCT
ERN1	CGGCCTCGGGATTTTTGGA	AGAAAGGCAGGCTCTTCCAC
XBP1s	CCTGGTTGCTGAAGAGGAGG	CCATGGGGAGATGTTCTGGAG
TP63	TTCGGACAGTACAAAGAACGG	GCATTTCATAAGTCTCACGGC
Vimentin	ACAACCTGGCCGAGGACATC	GACGTGCCAGAGACGCATTG

Primer sets for ChIP-qPCR analysis.

Gene	Forward	Reverse
ILEI-bs1	AGACACACAGACACTCACCCA	GCCTATTCTCCACTGACCATA
ILEI-bs2	CAATTTAATTTCTGCAACCACT	TTTTCTCAGGAGATTTCCTACA
TMTC3-bs-1	CTGGCAACCAGCGGAGGC	CCTTGGCGGAGTTGGGGA
TMTC3-bs-2	AGAGCCTACAGTTCACGACCG	GAGCGCCTTACCTCCTCTTCT

Supplementary table 2 The analysis of TMTC3 expression in transcriptome sequencing based on 86-paired ESCC tissues.

The correlation analysis between TMTC3 expression and clinicopathological features.

Variables	Total patients(n=86)	TMTC3 low expression (n=53)	TMTC3 high expression(n=33)	*p* value
Age (year)				0.157
＜60	28 (32.6)	14 (26.4)	14 (42.4)	
≥60	58 (67.4)	39 (73.6)	19 (57.6)	
Gender				0.090
Male	60 (69.8)	33 (62.3)	27 (81.8)	
Female	26 (30.2)	20 (37.3)	6 (18.2)	
Smoking				0.658
Yes	40 (46.5)	26 (49.1)	14 (42.4)	
No	46 (53.5)	27 (50.9)	19 (57.6)	
Alcohol				0.172
Yes	33 (38.4)	17 (32.1)	16 (48.5)	
No	53 (61.6)	36 (67.9)	17 (51.5)	
Histological grade				
Ⅰ	4(4.7)	3 (5.7)	1 (3.0)	0.612
Ⅱ	60 (69.8)	35 (66.0)	25 (75.8)	
Ⅲ	22 (25.6)	15 (28.3)	7 (21.2)	
TNM stage				**0.050**
Ⅱ and Ⅰ	61 (70.9)	42 (79.2)	19(57.6)	
Ⅲ	25 (29.1)	11 (20.8)	14 (42.4）	

Univariate and multivariate analysis by Cox Proportional Hazards model.

Variables	Univariate	*p* value	Multivariate	*p* value
	Hazard ratio(95% CI)		Hazard ratio(95% CI)	
TMTC3 expression (high vs. low)	**3.144 (1.520-6.378)**	**0.001**	**2.487 (1.122-5.509)**	**0.025**
Gender (male vs. female)	1.166 (0.536-2.537)	0.699	1.019 (0.402-2.583)	0.968
Age (< 60 vs. ≥ 60 years)	1.198 (0.508-2.474)	0.625	0.772 (0.347-1.718)	0.527
Smoking (yes vs. no)	0.758(0.371-1.547)	0.447	0.519 (0.135-1.996)	0.340
Drinking (yes vs. no)	1.211 (0.596-2.459)	0.597	1.850 (0.484-7.082)	0.369
Histological grade: Ⅲ vs. Ⅱ and Ⅰ	0.744 (0.329-1.682)	0.477	0.990 (0.41-2.389)	0.982
TNM stage: Ⅲ vs. Ⅱ and Ⅰ	**3.204 (1.574-6.524)**	**0.001**	**2.393 (1.117-5.126)**	**0.025**

Supplementary Figure Legends

Fig. S1

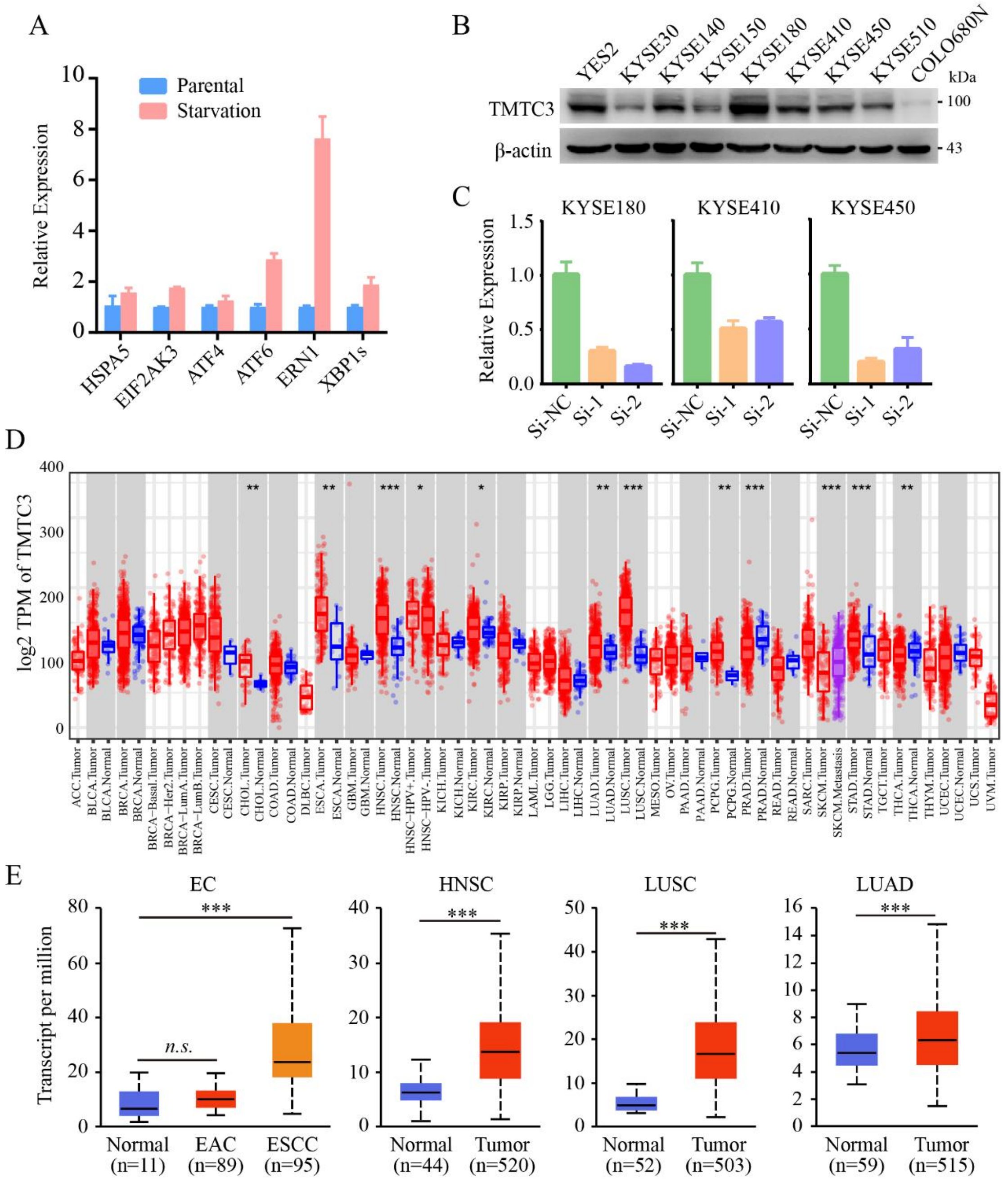

Supplementary Fig. S1 Expression of TMTC3 in various tumors from TCGA visible databases. A. The mRNA levels of ER sensors in KYSE450 cells after 24h cultured with amino-acid-free medium. B. Western blot analysis of TMTC3 in ten ESCC cell lines. C. The knockdown efficiency of TMTC3 by siRNA in KYSE180, KYSE410 and KYSE450 cells detected by qPCR. D. TMTC3 mRNA levels in various tumors from the TIMER2.0 database. E. Expression of TMTC3 in EC, HNSC, LUSC, LUAD from UALCAN database. EAC, esophageal adenocarcinoma. ESCC, esophageal squamous cell carcinoma. *n.s.*, not significant. ***, $p < 0.001$.

Fig. S2

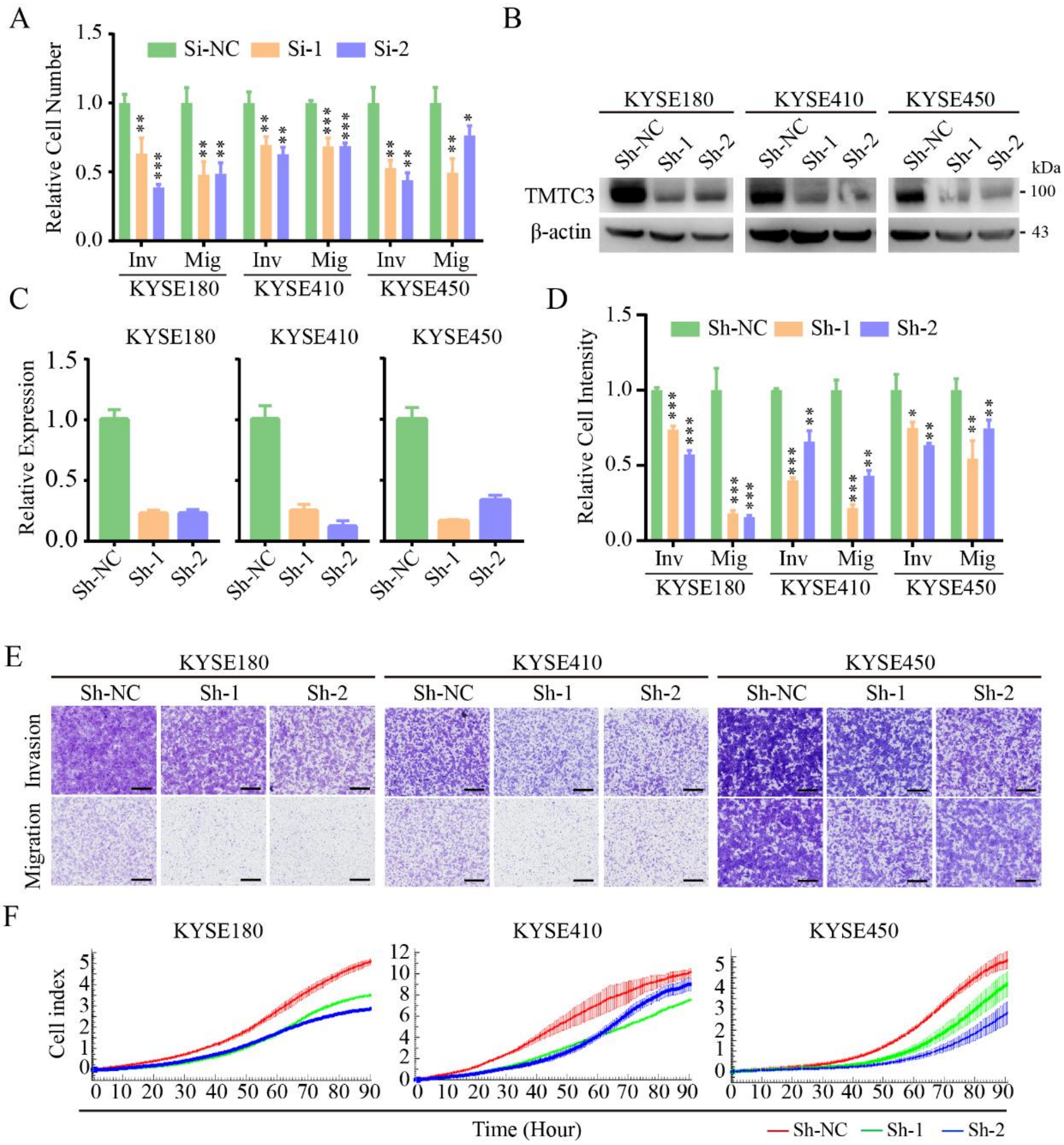

Supplementary Fig. S2 Knockdown of TMTC3 inhibited the malignant phenotype. A. Quantification results of Transwell assay in ESCC cells transfected with siRNA targeting TMTC3. B and C. The protein expression (B) and mRNA level (C) of TMTC3 in three cell lines stably expressing empty vectors and TMTC3-shRNA vectors. D and E. The quantification result (D) and representative images (E) of the Transwell assay in TMTC3 cells with stable knockdown. The scale bar represents 500 μm. *, $p < 0.05$. **, $p < 0.01$. ***, $p < 0.001$. F. Cell proliferation in TMTC3 cells stably knocked down via RTCA assay.

Fig. S3

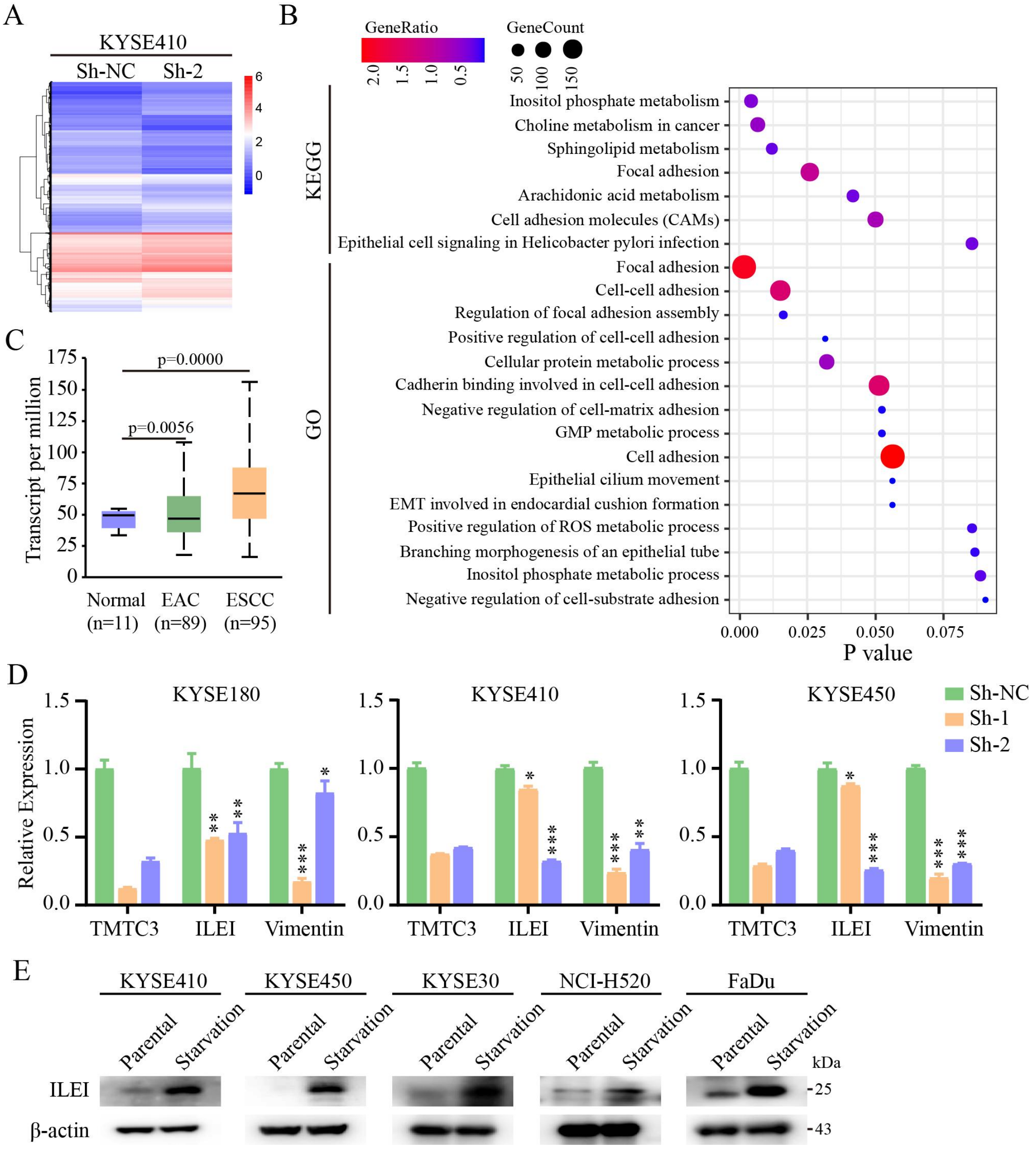

Supplementary Fig. S3 The expression of ILEI was inhibited in TMTC3 knockdown cells. A. Heatmap of DEGs by RNA-seq in KYSE410 cells stably infected with Sh-TMTC3 or Sh-NC. B. Pathway enrichment analysis for DEGs from (A). C. Statistical comparison of differences in the expression of ILEI in three groups from the UALCAN database. EAC, esophageal adenocarcinoma. ESCC, esophageal squamous cell carcinoma. D. The mRNA levels of ILEI and vimentin in Sh-TMTC3 cells. *, $p < 0.05$. **, $p < 0.01$. ***, $p < 0.001$. E. The expression of ILEI in SCCs cell lines after inducing ER stress.

Fig. S4

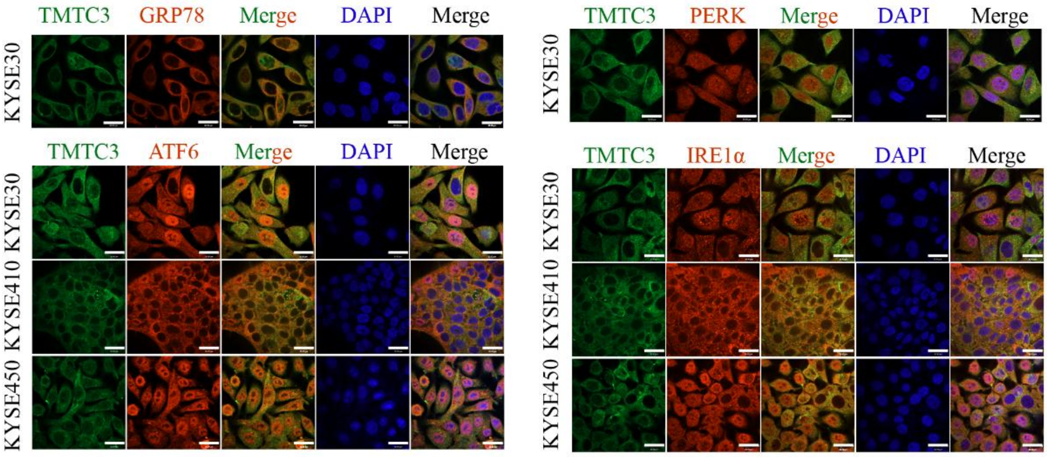

Supplementary Fig. S4 Colocalization of TMTC3 and GRP78, PERK, ATF6 or IRE1α in ESCC cell lines by IF. Scale bar, 30 μm.

Fig. S5

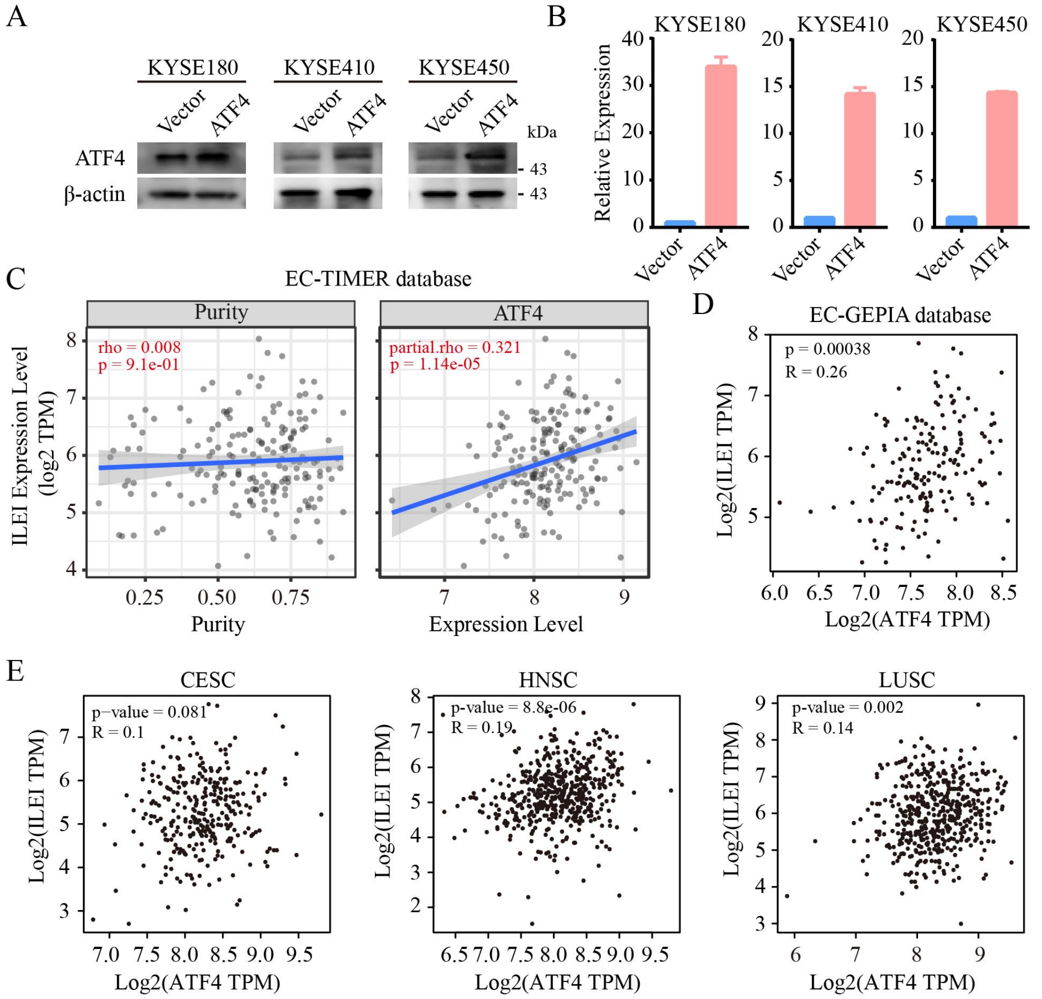

Supplementary Fig. S5 Positive correlation between ATF4 and ILEI. A and B. The overexpression of ATF4 at the protein level (A) and mRNA level (B). C and D. The correlation between ATF4 and ILEI in the TIMER2.0 (C) and GEPIA2 (D) databases. E. The Pearson's correlation coefficient between ATF4 and ILEI in other SCCs, including CESC, HNSC, LUSC.
